# Mastering the balance: BAK1’s dual roles in steering plant growth and immunity

**DOI:** 10.1093/hr/uhaf206

**Published:** 2025-08-01

**Authors:** Yujun Wu, Yonggui Ma, Wenying Wang, Shaoxiong Zhang, Wangze Wu

**Affiliations:** Key Laboratory of Medicinal Animal and Plant Resources of Qinghai-Tibetan Plateau in Qinghai Province, School of Life Sciences, Qinghai Normal University, Xining, China; Ministry of Education Key Laboratory of Tibetan Plateau Land Surface Processes and Ecological Conservation, Academy of Plateau Science and Sustainability, Qinghai Normal University, Xining, China; Ministry of Education Key Laboratory of Cell Activities and Stress Adaptations, School of Life Sciences, Lanzhou University, Lanzhou, China; Key Laboratory of Medicinal Animal and Plant Resources of Qinghai-Tibetan Plateau in Qinghai Province, School of Life Sciences, Qinghai Normal University, Xining, China; Ministry of Education Key Laboratory of Tibetan Plateau Land Surface Processes and Ecological Conservation, Academy of Plateau Science and Sustainability, Qinghai Normal University, Xining, China; Ministry of Education Key Laboratory of Tibetan Plateau Land Surface Processes and Ecological Conservation, Academy of Plateau Science and Sustainability, Qinghai Normal University, Xining, China; Key Laboratory of Medicinal Animal and Plant Resources of Qinghai-Tibetan Plateau in Qinghai Province, School of Life Sciences, Qinghai Normal University, Xining, China; College of Life Sciences, Northwest Normal University, Lanzhou, China

## Abstract

BAK1 was initially identified as a coreceptor of BRI1 in regulating the brassinosteroid-triggered signaling pathway in *Arabidopsis*. Over the past two decades, increasing pieces of evidence have demonstrated that BAK1 and its close paralogs form receptor–coreceptor complexes with distinct ligand-binding receptors. Through ligand-induced heterodimerization with receptor-like protein kinases or receptor-like proteins, BAK1 thereby regulates a variety of physiological events such as plant development, immunity, and stress responses. Thus, BAK1 plays a central role in directly governing the trade-offs of multiple signaling pathways. Deciphering the molecular mechanisms underlying how BAK1 coordinates plant growth and defense, with specific emphasis on its coreceptor functions, holds significant potential for future advancements in crop breeding. This review seeks to explore the latest insights into how BAK1 impacts the intricate equilibrium between plant development and immunity, as well as its roles in coordinating the activation of pattern-triggered immunity and effector-triggered immunity or programmed cell death. Furthermore, it offers significant perspectives on why BAK1 has been chosen as a shared component in determining plant growth and defense mechanisms across model plants to horticultural crops.

## Introduction

Most higher plants are sessile organisms that are constantly facing various abiotic and biotic stresses. During co-evolution, plants have evolved a diverse array of proteins referring to receptor-like protein kinases (RLKs) and receptor-like proteins (RLPs), which play essential roles in perceiving extracellular stimuli and initiating intracellular signals to precisely regulate plant growth, development, and stress adaptations. Within these cell surface-localized receptors, somatic embryogenesis receptor kinases (SERKs) typically function as common regulators shared by multiple signals [1]. Upon ligand-induced formation of heteromeric complexes, composed of coreceptor SERKs and specific receptor RLKs, transphosphorylation occurs to activate downstream signal components and initiate intracellular signaling events [2, 3]. Similar to most RLKs, SERKs are localized on the plasma membrane and possess an extracellular domain. Different from ligand-binding receptors that often contain large extracellular domains, the ectodomains of SERKs are relatively short, consisting of four to five leucine-rich repeats (LRRs), which are not responsible for direct ligand perception but are associated with various RLKs and RLPs to form new interaction surfaces for substrate docking. During this process, the ligand molecules function as molecular glue, facilitating the formation of heterodimers between the extracellular domains of RLKs/RLPs and BAK1. Within the cytosol, SERKs possess an intracellular kinase domain. After ligand binding, SERKs are activated and subsequently undergo interactive phosphorylation with the receptor, thereby initiating downstream signal transduction. Following the kinase domain, SERKs also possess a concise C-terminal tail, which is crucial for plant immunity and development differentiation [4]. Additionally, a single-pass transmembrane domain connects the extracellular domain of SERKs with the cytoplasmic region, thereby anchoring them to the plasma membrane.

Receptor kinase BRASSINOSTEROID INSENSITIVE 1 (BRI1)-ASSOCIATED RECEPTOR KINASE 1 (BAK1), also known as SERK3, was originally demonstrated as a coreceptor of the brassinosteroid (BR) receptor BRI1 to mediate phytohormone responses and regulate plant growth and development [5, 6]. Additional studies revealed that BAK1 and its closest homolog, BKK1/SERK4, are not only essential to the BR signal transduction but also critical to plant autoimmunity [7, 8]. BAK1, therefore, functions as a key modulator maintaining the balance between growth/development and biotic stress adaptations in plants [9–12]. Our better understanding of the detailed molecular mechanisms of BAK1 in balancing the trade-off between plant growth and plant defense will significantly benefit crop improvements in molecular breeding.

**Figure 1:**
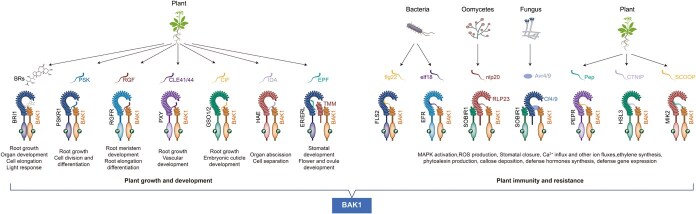
BAK1: a pivotal regulator balancing plant growth and immunity. BAK1 serves as a critical switch in the intricate interplay between plant growth and immunity. Upon recognition of various ligands by RLK or RLP, BAK1 is consistently recruited as a coreceptor, forming a specialized receptor complex that initiates signal transduction. The diversity of ligands leads to phosphorylation at different sites within BAK1, modulating its affinity with specific receptors. This dynamic interaction is crucial for maintaining the delicate balance between growth and defense mechanisms in plants.

### BAK1: orchestrating the growth-immunity equilibrium

*SERKs* were initially identified as cellular markers of forming somatic embryogenesis in carrot embryonic culture [13]. In *Arabidopsis*, *SERK* family belongs to the type II *LRR–RLKs* and consists of five members, *SERK1–SERK5* [9]. BAK1, also known as SERK3, was originally identified as a coreceptor of BRI1, the ligand-binding receptor for brassinosteroids (BRs), to regulate various aspects of plant development, such as seed germination, root growth, light response, stomata formation, and reproductive development [5, 6, 14]. Compared to strong growth and development defects observed in *bri1* null mutants, *bak1* only exhibited subtle BR defective phenotypes, raising the question whether BAK1 is essential to BR signal transduction or it merely acts to enhance the function of BRI1. Subsequent experiments indicated that under a dark-growing condition, the *serk1 bak1 bkk1* triple mutant displayed a phenotype similar to null *bri1* mutants, showing strong de-etiolated phenotype, featured as opened cotyledons and extremely shortened hypocotyls [7]. These findings provided solid genetic evidence demonstrating that BAK1 and its paralogs play an indispensable role in BRI1-mediated brassinosteroid signaling. Surprisingly, the simultaneously knock-out *BAK1* and *BKK1*/*SERK4* results in a spontaneous autoimmune response and a spontaneous cell death phenotype under the light-growing condition [8]. At the same time, an independent study discovered that two *bak1* alleles, *bak1-3* and *bak1-4*, exhibited increased susceptibility to various pathogens [15]. Furthermore, two simultaneously published studies pinpointed BAK1 as a coreceptor of FLAGELLIN-SENSING 2 (FLS2), playing an essential role in the perception of pathogenic flagellin [16, 17]. Indeed, plant disease resistance and BR-mediated growth represent opposing processes. However, BAK1 is implicated in both pathways. These unexpected findings give rise to an intriguing concept: BAK1 may function as a molecular switch that coordinates the balance between plant growth and immune responses.

Actually, besides the BR signaling pathway, an increasing number of studies have revealed the multifaceted roles of BAK1 in regulating plant growth and development. For instance, BAK1 forms heterodimers with PHYTOSULFOKINE (PSK) RECEPTOR 1 (PSKR1) and RGF1 INSENSITIVES (RGIs) to perceive PSK and RGF1, respectively, to modulate plant root growth [18, 19]. BAK1 associated with PHLOEM INTERCALATED WITH XYLEM (PXY) is instrumental in maintaining vascular stem cells and preventing their differentiation into xylem cells [20]. BAK1 interacts with HAESA (HAE) to manage organ abscission and collaborates with ERECTA subfamily (ERf), TOO MANY MOUTHS (TMM), and HAESA-LIKE 1 (HSL1) to orchestrate stomatal development [21–24]. Moreover, BAK1’s interaction with ERf is essential for the precise regulation of female gametophyte development and the maturation of the surrounding sporophytic integuments [25]. In addition, the association between BAK1 and GASSHO1 (GSO1) plays a pivotal role in maintaining the integrity of the embryonic cuticle [26] (Fig. 1).

Due to the particular structure of BAK1, it also functions as a coreceptor for an array of immune receptors to mediate the plant’s immune responses. BAK1 dynamically associates with the ELONGATION FACTOR-TU (EF-Tu) RECEPTOR (EFR), MALE DISCOVERER 1-INTERACTING RECEPTOR-LIKE KINASE 2 (MIK2), PEP1 RECEPTOR 1 (PEPR1), and PEPR2 to sense EF-Tu, SERINE-RICH ENDOGENOUS PEPTIDE (SCOOP), and Pep signals, respectively, which are known as pathogen-associated molecular patterns (PAMPs) or damage-associated molecular patterns (DAMPs) that are essential for the activation of immune signaling pathways [27–30]. Furthermore, BAK1 collaborates with SUPPRESSOR OF BIR1 1 (SOBIR1) and either RLP23 or RLP30 to perceive NECROSIS-AND ETHYLENE-INDUCING PEPTIDE 1 (NEP1)-LIKE PROTEINS (NLPs) and SCLEROTINIA CULTURE FILTRATE ELICITOR 1 (SCFE1), respectively, thereby conferring resistance to a range of pathogens [31, 32] (Fig. 1). Among other species, tomato LRR–RLP receptors Cf4 and Cf9 recruit BAK1 to perceive the pathogen-secreted cysteine-rich proteins Avr4 or Avr9, triggering the disease resistance responses [33]. Similarly, in tobacco, the LRR–RLP NbCSPR requires BAK1 to perceive bacterial cold shock protein csp22 and initiate defense responses [34]. As for rice, the recognition and immune response triggered by OsSSP1 (*Oryza sativa* secretory small protein 1) depend on an uncharacterized transmembrane OsSSR1 (secretory small protein receptor 1) and the key coreceptor OsBAK1 [35].

Given BAK1’s central role in mediating multiple signaling pathways associated with both development/growth and immunity, it is imperative for BAK1 to precisely distinguish and modulate these pathways to ensure reasonable resource allocation and optimal adaptation in plants. Therefore, when plants are exposed to different environments, the functional role of BAK1 will immediately undergo a corresponding change to balance the growth and immunity. However, the underlying mechanisms behind BAK1’s potent roles and the way it coordinates these two opposing processes remain elusive. Although current studies suggest that distinct ligand bindings can enhance the interactions between BAK1 and specific receptor proteins, it is noteworthy that the LRR domain, which is essential for interactions with various ligands and receptors, is highly conserved in BAK1 [36–38]. Hence, it is plausible to consider that binding affinities alone might not determine the specific functions of BAK1 in different signaling pathways.

The *bak1–5* mutant, bearing a C408Y mutation in the kinase domain of BAK1, exhibits significantly impaired kinase activity and markedly compromised immune activity, yet it maintains a wild-type-like morphology when it was crossed to *bkk1* [39]. This result implies that the variations in BAK1’s kinase activity and structural conformation could be responsible for its functional diversity. Some studies have demonstrated that phosphorylation sites, including T446, T449, T450, and T455, are pivotal in stabilizing the conformation of the key motifs of BAK1 [40, 41]. In particular, the T450A substitution rescued the seedling lethality observed in the *bak1–4 bkk1–1* double mutant that maintained complete insensitivity to flg22, implying that phosphorylation at this residue may specifically enhance BAK1’s regulation of FLS2 signaling [42]. Additionally, the T450A mutation partially suppressed the *bri1–5* phenotype [42], suggesting that this residue could be a critical mediator in BR signal transduction. Both the T449A and T446A mutants similarly reduce the overall autophosphorylation levels. However, these mutants retained most of their transphosphorylation activity toward FLS2 [40, 41]. This suggests that T446 and T449 play an important role in the recognition of specific BAK1 substrates. Researchers also examined the BAK1 phosphorylation sites mediated by flg22 and discovered that the S602/T603/S604 and S612 sites are necessary for BAK1 immune function. However, these phosphorylation sites are not necessary for BAK1-mediated BR-dependent plant growth regulation [43]. Based on those studies, it is reasonable to conclude that phosphorylation modifications at different sites of BAK1 may serve as key factors influencing its functional differentiation. SERINE/THREONINE PROTEIN PHOSPHATASE 2A (PP2A) and PP2C are types of protein phosphatases. Although it remains unclear whether PP2A or PP2C can influence plant growth and development by affecting BAK1 phosphorylation, studies have demonstrated that these protein phosphatases can negatively regulate BAK1 and thereby modulate innate immunity signaling [44, 45]. These results further indicate that the phosphorylation modification of BAK1 may play a certain role in coordinating plant growth and immune responses.

### BAK1: the immune switch for PTI–ETI synergy

Plants, as sessile organisms, have evolved a sophisticated two-layer immune system to combat the constant threats posed by pathogenic microorganisms. The first layer of plant innate immunity, known as pattern-triggered immunity (PTI), relies on the plasma membrane-localized pattern recognition receptors (PRRs), which can be RLKs or RLPs, to recognize pathogen-derived or plant-derived molecules, such as microbe-associated molecular patterns (MAMPs) or endogenous DAMPs. Upon ligand perception, PRRs undergo oligomerization to initiate cellular immune signaling and confer plants basal defense against a broad spectrum of pathogens [2]. Recent research has identified HAESA-LIKE 3 (HSL3) as an orphan receptor kinase capable of recognizing and binding novel DAMPs, CTNIPs, which facilitate interactions between HSL3 and BAK1 to initiate downstream immune responses [46]. In another DAMP-triggered immune pathway, BAK1 interacts with PEPR1 and PEPR2 to jointly mediate Peps-induced phosphorylation cascades [32, 47]. Interestingly, knock-out BAK1 unexpectedly amplifies PEPR-mediated signaling outputs while potentiating SA-dependent defense mechanisms [47]. These findings indicate that BAK1 not only acts as a coreceptor in plant immune response but also orchestrates defense responses through multilayered regulatory mechanisms encompassing both transcriptional reprogramming and post-translational modification networks. RLPs, similar to RLKs, rely on their extracellular structure to recognize PAMPs/DAMPs. However, unlike RLKs, RLPs lack cytoplasmic kinase domains, which means they cannot independently initiate intracellular signaling cascades. Instead, RLPs collaborate with RLKs, such as SOBIR1 and BAK1, using their distinct extracellular domains for ligand recognition, thereby activating immune responses [31, 48–50]. Despite this collaboration being well established, the specific downstream phosphorylation events between BAK1 and RLPs during immune response regulation remain poorly understood. To clarify BAK1’s role in immune signaling, structural biology approaches could be a valuable tool for probing the three-dimensional structures of PRRs and their interactions with ligands. This will help us gain a deeper understanding of the role of BAK1 in coordinating plant growth and immune signals activation.

In the ongoing co-evolutionary arms race between plants and pathogenic microorganisms, pathogens have developed highly effective biological molecules known as effectors. Effectors are usually injected into the cells by type III secretion system, attacking key PTI components to subvert immunity and enhancing their virulence. However, to counteract effector-mediated immunity suppression, plants have evolved intracellular nucleotide-binding domain LRR receptors (NLRs), which directly or indirectly recognize effectors and subsequently activate the second layer of plant innate immunity, known as effector-triggered immunity (ETI). Strong and profound responses such as a type of programmed cell death, hypersensitive response, are triggered by the initiation of ETI and often cause colocalized cell death and spontaneous lesion [51, 52]. Given the multifaceted roles of BAK1 in both growth and PTI signal transduction pathways, it is not surprising that pathogens target BAK1 as an ideal option. By interfering with BAK1, they can considerably undermine the plant’s immune response, thereby facilitating their own infection process. In this context, BAK1 assumes a pivotal role in the transition between PTI and ETI, allowing plants to sustain robust growth with minimal loss.

**Figure 2:**
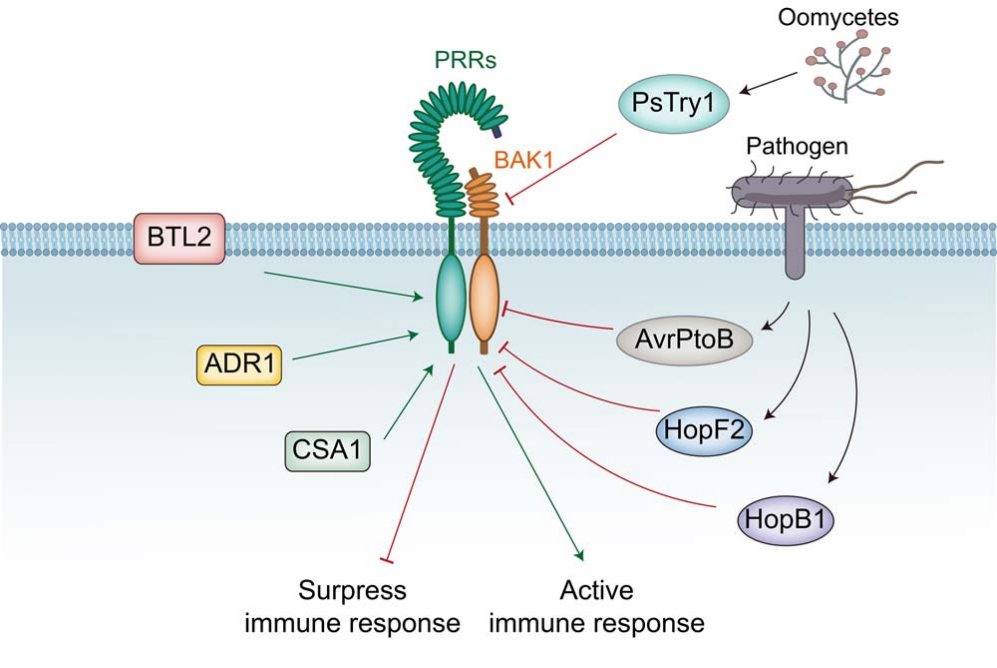
BAK1: a central node in plant immunity targeted by pathogen effectors and guardian proteins. BAK1 is targeted by pathogen-derived effector proteins such as AvrPtoB, HopF2, HopB1 and PsTry1, which have evolved to undermine BAK1’s function to suppressing plant immune responses. By disrupting BAK1, these effectors facilitate pathogen invasion by impairing the plant’s own defense mechanisms. Concurrently, guardian proteins such as ADR1, CHS1, and BTL2 play a pivotal role in activating plant immunity. They monitor the integrity of BAK1 to maintain immune homeostasis and trigger NLR-mediated cell death in response to pathogen attacks, thereby reinforcing the plant’s defenses. BAK1 serves as a shared signaling component in both PRR-mediated and NLR-mediated signaling pathways, highlighting the complex interplay between these two cascades. Its dual role is essential for the integration of plant immune responses and for preserving plant health under the stress of pathogen pressure.

Studies have demonstrated that the cell death phenotype observed in *bak1 bkk1* double mutant is a direct consequence of ETI activation. Moreover, mutation of the helper NLR proteins, ACTIVATED DISEASE RESISTANCE 1 (ADR1), can partially restore the autoimmune phenotypes of the *bak1 bkk1* genotype [53]. This suggests that under normal conditions, BAK1 and its paralogs are mainly responsible for maintaining the balance between plant growth and PTI. However, BAK1 is constantly guarded by NLR proteins. Once BAK1 is depleted or attacked by effectors, NLRs (such as ADR1s) can be constitutively activated, leading to spontaneous cell-death phenotypes and thus controlling pathogen invasion [53]. Overall, it can be concluded that BAK1 not only contributes to maintaining the balance between plant growth and immunity but also coordinates the timely initiation of PTI and ETI in the immune signaling pathway. CONSTITUTIVE SHADE AVOIDANCE 1 (CSA1), another NLR protein, does not physically interact with BAK1 but instead forms a complex with BIR3 to maintain the homeostasis of BAK1. Owing to biological or abiotic factors, when plants detect the absence of BAK1, they may rely on ADR1 and CSA1 to trigger immune response [54, 55]. Recently, a study has demonstrated that an uncharacterized receptor kinase BTL2 (BAK-TO-LIFE 2) monitors the integrity of BAK1. Upon detecting BAK1 depletion, BTL2 activates the Ca^2+^ channel CNGC20 (CYCLIC NUCLEOTIDE-GATED CHANNEL 20), in a kinase-dependent manner, to initiate plant autoimmunity [56]. Thus, the integrity of BAK1 is safeguarded by complex interplays among ADR1, CSA1, and BTL2 to effectively resist pathogenic bacterial infections through the ETI response (Fig. 2). Therefore, with BAK1 as the core, whether the presence or absence of BAK1 and its functional integrity may be the key to mediating the transition between PTI and ETI. Furthermore, it remains to be explored whether additional NLR proteins are involved in the surveillance of BAK1 and if specific phosphorylation sites of BAK1 are under NLR protein supervision.

**Figure 3:**
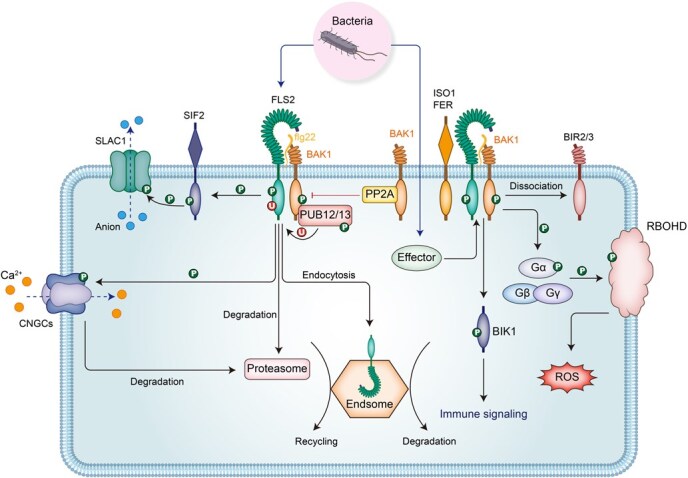
BAK1: a precise adaptor in plant immune signaling. The BAK1–FLS2 receptor complex serves as a paradigmatic example of the intricate regulation of plant immune signaling. Upon flg22 perception, FLS2 interacts with BAK1, setting off a phosphorylation cascade. Activated BAK1 then phosphorylates a diverse array of proteins, including RLK, RLCK, channel proteins, G proteins, and E3 ubiquitin ligases. This comprehensive phosphorylation event orchestrates the regulation of plant immune signal transduction. Concurrently, the activated FLS2–BAK1 complex fine-tunes immune responses by modulating endocytosis recycling or degradation pathways, as well as the ubiquitin–proteasome degradation pathway, ensuring a precise control of plant immune activation. Simultaneously, BAK1’s homeostasis is maintained through proteolytic processing, a conserved mechanism across species that is pivotal for plant immunity, growth, and the containment of cell death. The interplay between BAK1 and its interacting partners not only highlights the complexity of immune signaling but also underscores the importance of BAK1 as a central node in balancing plant growth and defense mechanisms.

In PTI signaling regulation, BAK1 has also been proven to function as a pivotal adaptor that orchestrates PTI activation through dynamic protein interactions. In the absence of pathogens, BAK1 is constitutively sequestered by BIR2 (BAK1-INTERACTING RECEPTOR-LIKE KINASE 2) and BIR3 via physical interactions, which prevents spontaneous dimerization between BAK1 and FLS2, keeping FLS2-mediated immune responses inactive to avoid autoimmunity [57, 58]. Notably, this negative regulatory mechanism extends to other RLKs, such as ANXUR1 (ANX1), a malectin-like domain-containing RLK, and BAK1-homologous NSP-INTERACTING KINASE 1 (NIK1), which use similar sequestration strategies [59, 60]. Conversely, IMPAIRED OOMYCETE SUSCEPTIBILITY 1 (IOS1) and the malectin-like receptor kinase FERONIA (FER) function as positive regulators that facilitate the formation of the FLS2–BAK1 complex. IOS1 primes the PTI response by interacting with FLS2 and BAK1 in a ligand-independent manner [61, 62], whereas FER acts as a scaffold protein to modulate receptor complex assembly upon ligand binding [63]. Together, these antagonistic regulatory systems collectively demonstrate that BAK1 operates as a signaling nexus integrating both positive and negative inputs to maintain plant growth and PTI equilibrium (Fig. 3). These results highlight two critical research frontiers: First, the spatiotemporal dynamics between competing regulatory complexes require systematic characterization. Second, the evolutionary logic underlying such intricate regulatory layers in PTI signaling demands deeper investigation. Resolution of these questions will provide fundamental insights into how plants optimize metabolic resources to reconcile growth-defense trade-offs, with potential applications in crop improvement strategies.

### Targeting of BAK1 by pathogen effectors

As previously mentioned, BAK1 is targeted by various pathogen effector proteins, which can impact both plant growth and immune responses.

AvrPtoB, a bacterial effector from *Pseudomonas syringae*, has been shown to interact with Pto, a protein kinase that plays a pivotal role in plant immunity in tomato [64]. Concurrently, Pto is known to associate with the resistance protein Prf. In the context of the presence of AvrPtoB, a conformational change within the Pto–Prf complex is triggered, which in turn activates Prf and initiates disease resistance response [65]. Structural analysis unveiled that AvrPtoB is equipped with two functional domains: the N-terminal region that is able to interact with multiple kinases, and the C-terminal region sharing a similar structure to E3 ubiquitin ligase [66, 67]. Due to the high structural similarities between the kinase domain of BAK1 and Pto, AvrPtoB can target and inhibit the kinase activity of BAK1 to suppress multiple immune signaling pathways triggered by PRRs [66] (Fig. 2). However, it is important to note that the C-terminus of AvrPtoB does not cause ubiquitination of BAK1 to attenuate plant immune resistance, suggesting that other targets of AvrPtoB ubiquitination are a subject worthy of further investigation. HopF2, another effector from *P. syringae*, directly interacts with BAK1 through its transmembrane and kinase domains, leading to the inhibition of plant immunity by suppressing BAK1 kinase activity [68]. Similarly, Xoo2875, an effector from *Xanthomonas oryzae*, associates with BAK1 in *O. sativa* to suppress immunity. However, the detailed mechanism underlying the interaction between Xoo2875 and BAK1 and its impact on plant immunity remain to be fully elucidated [69]. In contrast to HopF2 and AvrPtoB, HopB1, an effector with serine protease activity, cleaves activated BAK1 (Fig. 2). Interestingly, despite BAK1 being a target of HopB1, they do not interact with each other. Instead, HopB1 interacts with the FLS2 kinase domain in a flg22-independent manner [70]. Meanwhile, unlike AvrPtoB and HopF2 that significantly attenuate plant’s immune response, the HopB1-mediated enzymatic hydrolysis of BAK1 enhances plant defense activation [53, 70]. It has been confirmed that the kinase-dead mutants of BAK1 (BAK1 K317E and BAK1 D416N) and a phospho-site mutant of BAK1 (BAK1 T455A) display diminished response to HopB1-induced cleavage [70]. NIS1 (necrosis-inducing secreted protein 1) is a fungal effector initially identified in *Colletotrichum orbiculare* [71]. In recent years, researchers have analyzed the crystal structure of the NIS1 family proteins. Their studies have revealed that NIS1 exists as a β barrel composed of eight β strands, and the β4–β5 loop and β5 strand of NIS1 interact with the cytoplasmic region of OsBAK1, thereby inhibiting its kinase activity [71, 72]. Recently, in the pursuit of identifying pathogen effectors capable of suppressing cell death induced by the *Phytophthora* elicitin INF1, a novel apoplastic trypsin-like serine protease termed PsTry1 was discovered. This effector was found to specifically target the extracellular domain of soybean BAK1 and cleave it. By doing so, it effectively disrupts the assembly of the immune receptor complex, ultimately inhibiting plant PTI [73]. Nevertheless, it remains unclear which specific NLR proteins are implicated in the ETI response mediated by NIS1 and PsTry1, so elucidating this question will help us further understand the intricate battle between plants and pathogens (Table 1).

**Table 1:** Targeting of BAK1 by pathogen effectors.

**Effector protein**	**Pathogen source**	**Protein type**	**Mode of action**	**Target site**	**Reference**
AvrPtoB	Bacterium (*P. syringae* pv. tomato)	E3 ubiquitin ligase	Binds to the kinase domain of BAK1 and inhibits its activity	BAK1 kinase domain	[64], [66]
HopF2	Bacterium (*P. syringae* pv. tomato)	Unknown	Blocks the formation of BAK1–PRRs complex	BAK1 kinase domain	[68]
HopB1	Bacterium (*P. syringae* pv. tomato)	Serine protease	Cleaves activated BAK1	BAK1 phosphorylation site	[70]
NIS1	Fungus (*M. oryzae*, *Colletotrichum*)	β-Barrel structured protein	Inhibits BAK1 kinase activity	Cytoplasmic region of BAK1	[72]
PsTry1	Fungus (*Phytophthora*)	Serine protease	Cleaves the extracellular domain of BAK1	BAK1 extracellular domain	[73]

BAK1’s stability and homeostasis: the dynamic duo steering plant growth and immunity.

As reported, a Ca^2+^-dependent yet BAK1 kinase activity-independent proteolytic cleavage process can maintain the homeostasis of BAK1 [74]. The surface-exposed Asp (D287) residue of BAK1 is critical for its proteolytic cleavage and performs an essential role in BAK1-regulated plant immunity, BR responses, as well as cell death containment [74]. Mutation at this particular residue can severely compromise both the phosphorylation of BAK1’s substrate and the plasma membrane localization of BAK1, suggesting that the appropriate proteolysis of BAK1 protein plays a crucial role in regulating the timely activation of plant growth and immune signals [74]. Consequently, this offers a plausible explanation for the occurrence of a robust autoimmune response and cell death in plants when BAK1 has a loss-of-function mutation or is subjected to attack by pathogen effector proteins [2, 53]. On one hand, BAK1 orchestrates the balance between growth and PTI, without causing excessive consumption of limited resources to fight against invading pathogens. On the other hand, when plants encounter a strong pathogen attack that threatens their survival, BAK1 will sacrifice itself to activate the costly ETI signaling pathway, thereby forgoing plant growth and development to resist pathogen invasion and ultimately save the plant’s overall survival.

Interestingly, HopB1-induced cleavage occurs within the kinase domain between Arg-297 and Gly-298, which is distinct from the action of conserved eukaryotic proteases that cleave BAK1 at the transmembrane domain [60, 74]. In addition, the intrinsic protease-mediated BAK1 cleavage is independent of BAK1 kinase activity, yet bacteria or MAMP can trigger this progress. From this, it can be seen that the self-hydrolysis of BAK1 in plants and the protein hydrolysis mediated by pathogenic bacteria have fundamental differences. A question of interest arising from these findings is whether the hydrolysis of BAK1 triggers the production of different peptides by BAK1 itself, and whether those ligands can be recognized by different receptors to initiate an immune response in plants. However, regardless of whether such an unknown regulatory mechanism exists, analyzing the potential phosphorylation sites of BAK1 across different species may help explain how BAK1 can timely regulate the transition between growth and immunity.

Ca^2+^-permeable channel protein CNGC19/20, induced by BAK1 depletion, has been implicated in plant immunity and cell death [75]. Meanwhile, intracellular BAK1 homeostasis is also regulated through Ca^2+^-mediated proteolytic processing [74]. Additionally, the receptor kinase BTL2 and the NLR proteins ADR1 and CSA1 are known to safeguard BAK1 integrity or activity, triggering the ETI response [54–56, 71], and the activated ADR1, similar to the ZAR1 resistosome, forms a Ca^2+^ permeable channel that regulates plant immune responses through Ca^2+^ signaling [76, 77]. Since the cytoplasmic Ca^2+^ signature-triggered PRR or NLR activation is a key signal to initiate a series of downstream responses [78], a compelling question arising from these findings is how the Ca^2+^ signaling pathway is activated in the ETI pathway and whether BAK1 can be directly associated with those Ca^2+^-permeable channels to induce Ca^2+^ influx.

Unlike the mutations in its homolog *BIR1*, the mutations in *BIR3* do not independently cause cell death and immune responses [79, 80]. However, the *bak1 bir3* double mutant exhibits notable cell death and autoimmune phenotypes, similar to those observed in the *bak1 bkk1* double mutant. Moreover, the endogenous BAK1 levels in the *bir3* mutant are significantly reduced compared to wild-type plants [58, 80]. Those results highlighting the role of BIR3 differ from its homolog in regulating BAK1 stability, and suggest that plants employ various regulatory mechanisms to maintain appropriate BAK1 levels to balance the plant growth and immunity. Therefore, excessive accumulation of BAK1 in plants, or overexpression of the BAK1’s ectodomain in *Arabidopsis*, can also cause an imbalance between plant growth and immunity [74, 81, 82]. However, when the *OsBAK1* ectodomain is overexpressed in rice, the transgenic plants showed a phenotype similar to a wild-type plant, with no cell-death phenotype observed. By contrast, overexpression of the truncated intracellular domain of OsBAK1 resulted in a dwarfed phenotype, similar to the rice BR-insensitive mutant plants [83]. This phenotype is mainly due to the significantly suppressed expression levels of *OsBAK1* in these transgenic lines. However, upon overexpression of full-length *OsBAK1*, the transgenic rice plants exhibited completely different phenotypes, such as higher yield as well as corrugated and twisted leaf phenotypes [84]. Moreover, heterologous overexpression of *ZmBAK1* from maize in rice also results in increased plant height, weight, and yield [85]. These findings further indicate that the stability and homeostasis of BAK1 serve as a foundation for ensuring its proper membrane distribution, thereby preventing excessive activation or inhibition of growth and immunity signaling. Meanwhile, it appears that the function of BAK1 exhibits a certain degree of differentiation across different crops [85]. To date, although many studies have confirmed the role of BAK1 in regulating plant growth and immunity in crops, there are still significant gaps in the interpretation of its molecular mechanism. Therefore, exploring the evolutionary history of BAK1 in crops and analyzing the molecular basis of its functional divergence across different species could offer crucial insights for developing high-yielding and disease-resistant crops.

It is known that bacteria or MAMP-activated FLS2 relies on BAK1 and its kinase activity, and FLS2 undergoes internalization through endocytosis or ubiquitination for degradation to maintain that the growth and immune signaling pathways are not excessively activated [38, 86, 87]. However, it remains unclear in plant immunity signaling, whether BAK1 itself can undergo endocytosis or ubiquitination-mediated degradation pathways to balance the plant growth and immunity. But in the BR signaling pathway, MSBP1 (MEMBRANE STEROID-BINDING PROTEIN 1) interacts with the extracellular domain of BAK1 to accelerate BAK1 endocytosis, which results in suppressed BR signaling by shifting the equilibrium of BAK1 toward endosomes [88]. Meanwhile, BR treatment increases the SUMOylation level of BAK1, and specifically affects the interaction between BAK1 and BRI1 without affecting its interaction with PEPR [89]. In the future, by leveraging proteomics technologies to analyze the differential proteins interacting with BAK1 in crops before and after pathogen treatment, we may gain valuable insights into the BAK1-induced plant immunity and development regulation.

## Conclusion and perspective

From an evolutionary perspective, BAK1 is a highly conserved and ancient protein kinase found across a diverse range of plant species, including monocots, dicots, and nonvascular plants [90]. This conservation under evolutionary pressure implies that BAK1 is selected for its unique protein structures that enable it to function as a coreceptor in various signaling pathways [91]. As a pivotal decision-making protein, BAK1 is essential for mediating growth and immune signaling pathways in plants by associating with distinct receptors as well as regulating its own activity or abundance. Nevertheless, whether other ancient, evolutionarily conserved receptor kinases with shorter extracellular domains, such as CLAVATA3 INSENSITIVE RECEPTOR KINASES (CIKs), can also function as coreceptors in multiple signaling pathways is an open question that needs further investigation. Answering this question might also help to reveal the functional diversity exhibited by BAK1.

The ligand molecules function as molecular glue, promoting the formation of specific receptor complexes and thereby regulating specific biological processes. In this process, BAK1 is essential not only for enhancing ligand binding but also for initiating downstream immune signaling. However, to date, only a limited number of ligand molecules and their corresponding receptors have been identified, particularly in horticultural crops. Therefore, based on the current construction of protein structure models and the simulation of protein molecule docking, it will greatly facilitate the exploration of new ligand–receptor pairs. Last year, researchers systematically analyzed the structure of the NIS1–BAK1 protein complex. Utilizing a DNA-encoded compound library, they successfully screened and identified the small molecule compound B156, which can bind to both NIS1 and BAK1 simultaneously. Moreover, the researchers discovered that this particular compound is capable of blocking the binding of NIS1 and BAK1, thereby inhibiting the virulence of *Magnaporthe oryzae* on rice [72]. This groundbreaking research lays a robust theoretical foundation and offers a novel research avenue for the future development of new biopesticides.

Furthermore, artificial intelligence has already significantly advanced the life sciences research. Most notably, AlphaFold has enabled highly accurate prediction of previously elusive protein structures [92]. This will facilitate the analysis of the crystal structure of the BAK1 protein and the interaction receptors. Nevertheless, research on the molecular mechanism of BAK1 in regulating growth and immunity in crops is still relatively lagging behind. Many of the regulatory mechanisms still require further in-depth analysis. Moreover, there is still lack of studies concerning the evolution of BAK1 across different species as well as the functional research on specific sites of BAK1 in crops. This situation poses significant obstacles to our future endeavors to produce crop plants with broad-spectrum resistance and without growth penalty through various approaches, such as gene knock-outs (including mutations or deletions), knock-ins (like insertions), knock-up/knock-down, or site-specific mutations.

## Data Availability

No datasets were generated or analyzed during the current study.
